# Ras-related protein Rab-20 inhibition alleviates cerebral ischemia/reperfusion injury by inhibiting mitochondrial fission and dysfunction

**DOI:** 10.3389/fnmol.2022.986710

**Published:** 2022-10-25

**Authors:** Jia Guo, Lu Zhang, Yujie Bu, Wenjuan Li, Jianping Hu, Jianxiong Li

**Affiliations:** Department of Neurology, Lanzhou University Second Hospital, Lanzhou, Gansu, China

**Keywords:** Rab20, cerebral ischemia/reperfusion, mitochondria, mitochondrial fission, apoptosis

## Abstract

Ras-related protein Rab-20 (Rab20) is induced in hypoxia and contributes to hypoxia-induced apoptosis. However, the role and mechanism of Rab20 in cerebral ischemia/reperfusion (I/R) injury need to be elucidated. We established a cerebral I/R injury model in the mice and an oxygen-glucose deprivation/reoxygenation (OGD/R) model in HT22 cells to determine the effects of Rab20 in cerebral I/R injury. Rab20 expression was upregulated in mice after I/R and in HT22 cells after OGD/R. Upregulated Rab20 was mainly located in neurons. Rab20 inhibition significantly alleviated brain infarct volume, neurological deficits, and neuronal apoptosis in mice after I/R. Moreover, Rab20 knockdown significantly ameliorated the OGD/R-induced inhibition of cell viability and apoptotic cell death in HT22 cells. Rab20 knockdown significantly alleviated OGD/R-induced mitochondrial fission by repressing mitochondrial dynamin-related protein 1 (Drp-1) recruitment and increasing Drp-1 (Ser637) phosphorylation and ameliorated mitochondrial dysfunction by reducing the mitochondrial reactive oxygen species (ROS) and cellular calcium accumulation and increasing the mitochondrial membrane potential. In addition, Rab20 knockdown significantly alleviated cytochrome c release from the mitochondria into the cytosol in HT22 cells after OGD/R. Rab20 contributes to cerebral I/R injury by regulating mitochondria-associated apoptosis pathways. Targeting Rab20 may be an attractive strategy for the treatment of cerebral I/R injury.

## Introduction

Stroke, a cerebrovascular accident, is a leading cause of mortality and long-term disability in adults (Campbell et al., 2019). Ischemic stroke is the major type that is caused by the sudden blockage of blood flow, leading to brain tissue injury due to reduced blood and oxygen supply (Gąsecki et al., 2021). At present, the primary therapeutic approach for ischemic stroke treatment is immediate recanalization of the occluded artery and reperfusion of the brain tissue. Intravenous thrombolysis is the only FDA-approved drug for patients with cerebral ischemia. Although its use is limited by a narrow therapeutic window (within 4.5 h of symptom onset) and hemorrhagic complication, this treatment is the most effective for patients with cerebral ischemia (Prabhakaran et al., 2015; Liu et al., 2018). However, reperfusion causes accelerated neuronal damage due to a significant increase in oxidative stress and inflammation (Lin et al., 2016). Reperfusion injury has become a critical challenge in stroke treatment. Thus, it is importance to understand the mechanisms of ischemia/reperfusion (I/R) injury in the brain to develop effective therapeutics.

After ischemic stroke, the depletion of blood and oxygen supply causes the release of glutamate into the extracellular space, thereby producing an influx of calcium into the cell, which can rapidly induce neuronal death in the ischemic core, and the damage is irreversible (Hermann et al., 2001; Zhou et al., 2018). However, I/R-induced neuronal loss in the transition or penumbra zone is not irreversible (Zhou et al., 2018). Although I/R-induced neuronal death in the penumbra zone is not usually lethal, it contributes substantially to the loss of neurologic function and cognitive deficits (Hermann et al., 2001). Thus, reducing neuronal apoptosis in the ischemic penumbra is a potential treatment method for I/R injury.

Ras-related protein Rab-20 (Rab20) belongs to the Rab subfamily of small GTPases. Rab20 was first reported to be located in apical dense tubules which contribute to apical endocytosis/recycling (Lutcke et al., 1994). Rab20 plays an important role in immune regulation by controlling endosome maturation in macrophages (Egami and Araki, 2012; Zhao et al., 2020). In retinal endothelial cells and retinal Müller cells, high glucose induced Rab20 expression, and upregulated Rab20 contributed to high glucose-induced cell apoptosis (Kim et al., 2020). In a hypoxic microenvironment, Rab20 is directly induced by hypoxia-inducible transcription factor 1 (HIF-1) and is involved in hypoxia induced apoptosis (Hackenbeck et al., 2011). Moreover, Rab20 expression was substantially upregulated in an experimental model of brain inflammation in mice (Liang et al., 2012). Hypoxia and inflammation are important causes of I/R injury. Therefore, these findings suggested that Rab20 may play an important role in I/R injury.

In this study, we determined the role and mechanisms of Rab20 in I/R injury. Rab20 expression was significantly elevated in mice after I/R and in HT22 cells after oxygen-glucose deprivation/reoxygenation (OGD/R). Rab20 knockdown significantly alleviated brain infarct volume, neurological deficits, and neuronal apoptosis by inhibiting mitochondria-associated apoptosis pathways. Rab20 may be a novel target gene for the treatment of the cerebral I/R injury.

## Materials and methods

### Experimental animals

A total of 120 male C57BL/6J mice, 6–8 weeks old, were purchased from the Lanzhou University Second Hospital. Mice were housed in a specific pathogen-free (SPF) animal facility with a 12 h light/12 h dark cycle and given free access to water or food. Animals for each group were randomized. All animal procedures were approved by the Ethics Committee of Lanzhou University Second Hospital (D2020-046). Animal experiments were conducted in accordance with the guidelines of the Institutional Animal Care and Use Committee of the Institute of Nutrition and Health.

### Construction of middle cerebral artery occlusion and reperfusion models and treatment

Ischemia/reperfusion surgery were performed on male C57BL/6J mice using the intraluminal filament method as described previously (Liu et al., 2003, 2019). Briefly, mice were anesthetized with ketamine (12 mg/kg) and xylazine (10 mg/kg) by intramuscular injection. The neck was depilated, and the right common carotid bifurcation was exposed. To induce stroke, a silicone-coated 8-0 filament was inserted to the internal carotid artery to occlude the origin of the right middle cerebral artery for 1 h. The regional cerebral blood flow during surgery was measured with a laser Doppler flowmetry (PeriFlux System 5000; Perimed, Stockholm, Sweden). A reduction of 80% in the cerebral blood flow during surgery was considered successful. After 1 h of occlusion, the filament was removed to recover the cerebral blood flow. In the sham group, right carotid arteries of mice were surgically exposed and the suture was not inserted.

To knockdown the expression of Rab20, adeno-associated virus (AAV) vectors were constructed. AAV9-Syn-GFP-U6-shRab20 (shRab20) and AAV9-Syn-GFP-U6-negative control (shNC) were constructed by Sunbio Medical Biotechnology (Shanghai, China). The shRab20 and shNC sequences inserted in the AAV9 vector were shown in Table 1. After concentrating, the shRab20 and shNC AAV particles were diluted in PBS to 1 × 10^11^ genome copies/100 μl. AAV particles containing either shRab20 or shNC were stereotactically injected into the right lateral ventricle (bregma: −2.2 mm, dorsoventral: 3 mm, lateral: 1 mm) for 4 weeks prior to middle cerebral artery occlusion (MCAO) operation (Zhao et al., 2013; Choi et al., 2015). A total of 3 × 10^9^ genome copies of shRab20 or shNC AAV virus in 3 μl were injected into each animal at a rate of 200 nl/min (Jin et al., 2019).

**TABLE 1:** shRNA, siRNA, and primers.

Name	Sequence
shRab20	5′-CCGGGAAGATCCTGAAGTACAAGATCTCGAGATCTTGTACTTCAGGATCTTCTTTTTT-3′
shNC	5′-CCGGTTCTCCGAACGTGTCACGTCTCGAGACGTGACACGTTCGGAGAATTTTTT-3′
siRab20	5′-GAAGAUCCUGAAGUACAAGAUTT-3′
siNC	5′-UUCUCCGAACGUGUCACGUTT-3′.
Rab20 Fwd	5′-TCTCCACAGGTACCAAG-3′
Rab20 Rev	5′-CCACAGTCAACAAGTT-3′
β-Actin Fwd	5′-GTGACGTTGACATCCGTAAAGA-3′
β-Actin Rev	5′-GCCGGACTCATCGTACTCC-3′

### Neurological score

Neurobehavioral score was counted at 72 h after I/R, as described previously (Jin et al., 2015). In a typical procedure, neurobehavioral evaluation was determined according to the following scoring system: 0, no deficit; 1, forelimb flexion; 2, the same as 1, plus decreased resistance to lateral push; 3, unidirectional circling; 4, longitudinal spinning or seizure activity; and 5, no movement.

### Brain infarction measurement

Infarct volume was determined by 2,3,5-triphenyltetrazolium chloride (TTC) staining at 72 h after I/R. Mice were anesthetized. The brain was removed from each animal and cut into 2 mm-thick slices. The slices were incubated with 2% TTC solution (Coolaber, Beijing, China) at 37°C for 30 min. Finally, the slices were fixed in 4% paraformaldehyde overnight and the infarction volume was quantified using ImageJ software (National Institutes of Health, Bethesda, MD, USA).

### Immunofluorescence

Mice were anesthetized at 72 h after I/R and perfused with PBS and 4% paraformaldehyde. The brain was removed from each animal and fixed with 4% paraformaldehyde overnight, transferred to 20 and 30% sucrose, and cut into 5 μm-thick sections on a freezing microtome. After washing and blocking, the sections were incubated overnight at 4°C with the following primary antibodies: anti-Rab20 (1:200; 11616-1-AP, Proteintech Group, Inc., Wuhan, China), anti-NeuN (1:100; 66836-1-Ig, Proteintech Group, Inc.), anti-IBA1 (1:500; 011-27991, Fujifilm Wako, Japan), and anti-GFAP (1:200; ab279290, Abcam, Cambridge, MA, USA). After washing, the sections were incubated with donkey anti-rabbit Alexa fluor 488 secondary antibody (1:500; ab150073, Abcam), donkey anti-rabbit Alexa fluor 555 secondary antibody (1:500; A31572, Thermo Fisher Scientific, MA, USA), donkey anti-mouse Alexa fluor plus 555 secondary antibody (1:500; A32773, Thermo Fisher Scientific, MA, USA), and donkey anti-goat Alexa fluor plus 555 secondary antibody (1:500; A32816, Thermo Fisher Scientific) for 2 h at room temperature in the dark. Finally, the nuclei were stained by 4′,6-diamidino-2-phenylindole (DAPI) for 15 min.

### Terminal deoxynucleotidyl transferase-mediated dUTP nick end labeling staining

For terminal deoxynucleotidyl transferase-mediated dUTP nick end labeling (TUNEL) staining in brain tissues, the sections were stained with TUNEL solution (Roche Diagnostics, Indianapolis, IN, USA) for 1 h at 37°C. After washing, the neurons were stained with anti-NeuN (1:100; 66836-1-Ig, Proteintech Group, Inc.) for 1 h at 37°C. The sections were incubated with donkey anti-mouse IgG (1:100; 715-685-150, Jackson ImmunoResearch Inc., West Grove, PA, USA). The sections were photographed using a microscope with a digital camera (Olympus, Tokyo, Japan). Both TUNEL (red) and NeuN (blue) positive cells were counted as the apoptotic neurons. Five different fields were taken from the ischemic penumbra of each section using 40 × objective lens (Zhang et al., 2013; Liu et al., 2020). Eight consecutive sections per mouse and six mice per group were analyzed. The percentages of TUNEL-positive neurons relative to neurons were counted by an investigator who was blinded to the experimental groups.

HT22 cells after treatment were fixed in 4% paraformaldehyde for 30 min at room temperature. After washing three times with PBS, the cells were incubated in 0.3% Triton X-100 for 5 min. Then, the cells were incubated with TUNEL solution (Roche Diagnostics, Indianapolis, IN, USA) for 1 h at 37°C and DAPI was used to stain the cell nuclei. TUNEL-positive cells were observed under a light microscope (Olympus, Tokyo, Japan). Both TUNEL (red) and DAPI (blue) positive cells were counted as the apoptotic cells. Average number of three images under a fluorescence microscope at 200× magnification from each treatment group was presented as the final results, and each group was repeated three times independently.

### Oxygen-glucose deprivation/reoxygenation model

HT22 is an immortalized mouse hippocampal neuron cell line and was purchased from Procell Life Science & Technology Co., Ltd. (Wuhan, China). Cells were cultured in DMEM (Gibco, Carlsbad, CA, USA) containing 10% FBS (Gibco, Carlsbad, CA, USA) at 37°C. HT22 cells were cultured in glucose-free DMEM solution supplemented with 100 U/ml penicillin and 100 mg/ml streptomycin and placed into a hypoxic chamber (Thermo Fisher Scientific Inc.) with 1% O_2_, 5% CO_2_, and 94% N_2_ for 0, 1, 2, 4 or 8 h to mimic OGD (Zhang et al., 2021). After OGD, cells were given normal DMEM with 10% FBS for 24 h.

### Cell transfection

Ras-related protein Rab-20 siRNA (siRab20) and scramble negative control siRNA (siNC) were synthesized by GenePharma (Shanghai, China). The siRab20 and siNC sequences were shown in Table 1. HT22 cells were transfected using Lipofectamine^®^ 2000 Reagent (Invitrogen, Carlsbad, CA, USA) according to the manufacturer’s instructions.

### Cell viability assay

Cell Counting Kit-8 (CCK-8) assay was used to analyze the cell viability of HT22 cells according to the manufacturer’s instructions. HT22 cells were cultured on 96-well plates at a density of 1 × 10^4^ cells/well for 24 h and treated with OGD/R. CCK-8 solution (Beyotime, Shanghai, China) at 10 μl was added to each well. Cells were then incubated for 1 h at 37°C. The optical density (OD) value of each well was measured at 450 nm using an automatic microplate reader (Bio-Tek M200, Tecan, Austria).

### Real-time quantitative polymerase chain reaction

Total RNA was isolated from brain tissues and HT22 cells using TRIzol reagent (Invitrogen) according to the manufacturer’s protocol. The quantity and integrity of total RNA were analyzed by nanodrop spectrophotometer and gel electrophoresis. Total RNA was reverse transcribed into cDNA by M-MLV RTase (Promega, Madison, WI, USA). The Rab20 mRNA expression was determined using SYBR Master Mixture (TAKARA, Dalian, China). The 2^–ΔΔ^ *^Ct^* analysis method with normalization to β-actin expression was used to calculate the relative expression of Rab20. Primers used in this study were shown in Table 1.

### Western blot

Brain tissues and HT22 cells were lysed using RIPA lysis buffer (Beyotime) containing proteinase inhibitor to obtain the whole cell proteins. For the analysis of mitochondrial proteins from cells, a Cell Mitochondria Isolation Kit (Beyotime) was used to isolate mitochondria according to the manufacturer’s instruction. Mitochondria were lysed with a cold mitochondrial protein extraction kit (KeyGEN Biotech, Nanjing, China) to obtain mitochondrial proteins. The concentrations of the total proteins were measured by a BCA protein assay kit (Beyotime). Protein samples were denatured and separated by 10% SDS-PAGE. After SDS-PAGE, proteins were transferred to PVDF membrane. The PVDF membranes were then blocked with PBS containing 7.5% non-fat milk and incubated overnight with the primary antibodies at 4°C as follows: anti-Rab20 (1:1,000; 11616-1-AP, Proteintech Group, Inc.), anti-β-actin (1:2,000; 20536-1-AP, Proteintech Group, Inc.), anti-Bcl-2 (1:2000; 26593-1-AP, Proteintech Group, Inc.), anti-Bax (1:2,000; 60267-1-Ig, Proteintech Group, Inc.), anti-COX-4 (1:5,000; 11242-1-AP, Proteintech Group, Inc.), anti-Drp1 (1:2,000; 12957-1-AP, Proteintech Group, Inc.), anti-p-Drp1 (ser637; 1:1,000; ab193216, Abcam), and anti-cytochrome c (Cyto c; 1:4,000; 10993-1-AP, Proteintech Group, Inc.). After washing, membranes were incubated with HRP-conjugated anti-rabbit or anti-mouse secondary antibodies for 1 h. Immunoreactivity was detected using enhanced chemiluminescence reagents (Pierce Biotech, IL, USA).

### Mitochondrial fission assay

For mitochondrial fission assay, HT22 cells were cultured in confocal dishes. After treatment, cells were washed and incubated with 200 nM MitoTracker^®^ Deep Red FM (Yeasen, Shanghai, China) for 30 min at 37°C. Images were obtained by a confocal microscope (LSM 750, Zeiss, Gottingen, Germany).

### Mitochondrial membrane potential (Δψm) assay

For mitochondrial membrane potential (Δψm) assay, HT22 cells were grown on glass-bottom dishes. After treatment, cells were washed and incubated with tetramethylrhodamine ethyl ester perchlorate (TMRE, Beyotime) for 30 min at 37°C. Images were obtained by a confocal microscope (LSM 750, Zeiss).

### Mitochondrial reactive oxygen species production

For mitochondrial reactive oxygen species (ROS) assay, HT22 cells were grown on glass-bottom dishes. After treatment, cells were washed and incubated with 100 nM MitoTracker^®^ Green FM (Yeasen) and 5 μM MitoSOX Red Mitochondrial Superoxide Indicator (Yeasen) for 10 min at 37°C, as previously described (Wu et al., 2017).

### Intracellular calcium assay

For intracellular calcium assay, HT22 cells were grown on glass-bottom dishes. After treatment, cells were washed and loaded with 2.5 μM Fluo-3AM (Beyotime) in the dark for 30 min. After washing, a confocal microscope (LSM 750, Zeiss) was used to obtain images, and the fluorescent intensity was analyzed using ImageJ software (National Institutes of Health, Bethesda, MD, USA) (Liao et al., 2018).

### Statistical analysis

Three or more independent experiments were performed for all experiments. Data analyses were blinded by using different investigators. All the data are presented as mean ± SD. SPSS 19.0 software (IBM Corp., Chicago, IL, USA) was used for data analysis. The comparisons between groups were analyzed using Student’s *t*-test or one-way ANOVA. *P* < 0.05 was considered to be statistically significant. GraphPad Prism 5 software (GraphPad Software Inc., San Diego, CA, USA) was used for statistical graphing.

## Results

### Ras-related protein Rab-20 expression is significantly increased in mice after ischemia/reperfusion

**FIGURE 1:**
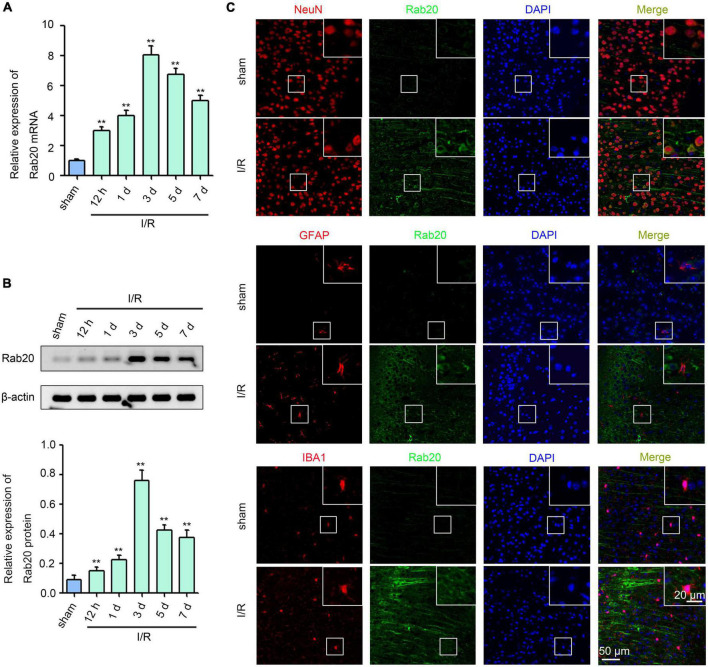
Ras-related protein Rab-20 expression was significantly increased in mice after I/R. **(A)** Rab20 mRNA levels were determined by RT-qPCR after I/R (*n* = 6). **(B)** Representative Western blot images and quantitative analyses of Rab20 protein in mouse brain after I/R (*n* = 6). **(C)** Double immunofluorescence staining for Rab20 (green) in neuron (NeuN, red), astrocytes (GFAP, red), and microglia (IBA1, red) in the penumbra after I/R. Scale bar, 20 and 50 μm. **P < 0.01 vs. the sham group.

Ras-related protein Rab-20 expression levels were determined at 12 h, 1 day, 3 days, 5 days, and 7 days in mice after I/R using real-time quantitative polymerase chain reaction (RT-qPCR) and Western blot. As shown in Figures 1A,B, Supplementary Table 1, and Supplementary Data Sheet 1, Rab20 mRNA and protein levels were significantly elevated at 12 h, peaked at 3 days, and decreased at 5 days after I/R compared with the sham group. Double-label immunofluorescence staining was performed to analyze the cellular localization of Rab20 in the penumbral area of cortex at 3 days after I/R. The Rab20 was significantly increased in neurons. Rab20 was mainly expressed in neurons (NeuN) in mice after I/R and not in astrocytes (GFAP) and microglia (IBA1) (Figure 1C).

### Ras-related protein Rab-20 knockdown ameliorated the functional outcomes after cerebral ischemia/reperfusion

**FIGURE 2:**
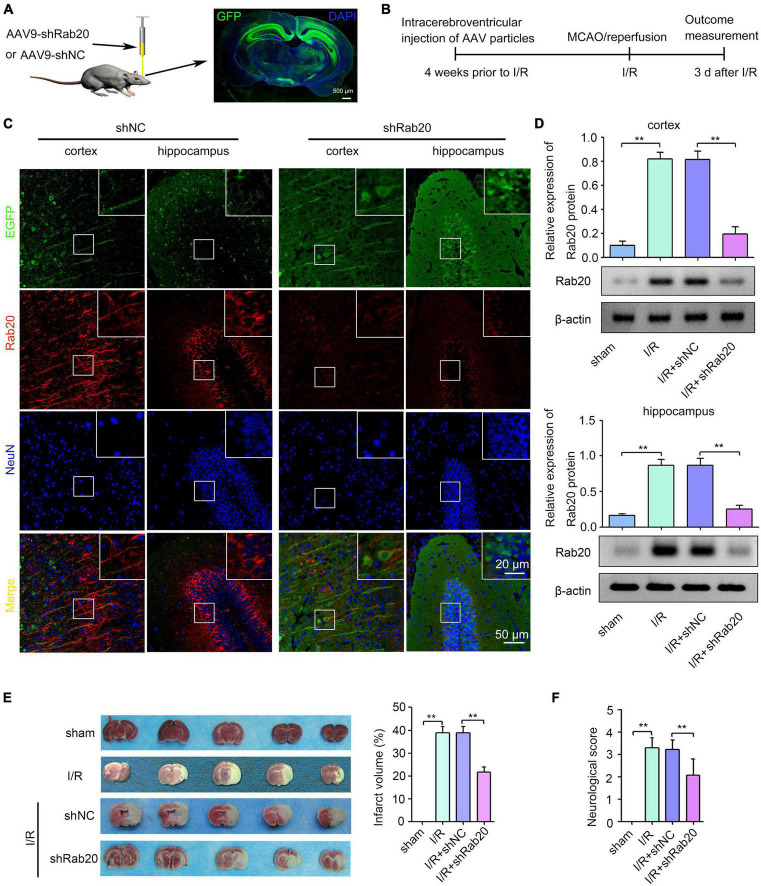
Ras-related protein Rab-20 knockdown significantly reduced the cerebral infarction and ameliorated the neurological outcome after cerebral I/R. **(A)** The shRab20 and shNC AAV particles were stereotactically injected into the right lateral ventricle for 4 weeks prior to MCAO operation. Scale bar, 500 μm. **(B)** Diagram of the experimental procedure. **(C)** The silencing efficiency of the shRab20 AAV particles in the penumbra of the cortex and hippocampus was determined by double immunofluorescence staining for Rab20 (red) in neuron (NeuN, blue) at 3 days after I/R. Scale bar, 20 and 50 μm. **(D)** Western blot assay was used to confirm the silencing efficiency of the shRab20 AAV particles in the penumbra of the cortex and hippocampus at 3 days after I/R (*n* = 6). **(E)** Representative photographs of coronal brain sections stained by TTC showing decreased infarct volume in shRab20-treated mice as compared to the shNC-treated mice at 3 days after I/R (*n* = 6). **(F)** Neurological scores were used to evaluate the neurological function at 3 days after I/R (*n* = 18). **P < 0.01.

To determine the role of Rab20 during cerebral I/R injury, we administrated shRab20 AAV particles into the right lateral ventricle for 4 weeks prior to MCAO operation (Figure 2A). As shown in Figure 2A, a robust GFP signal was observed at 4 weeks after AAV infection. After I/R for 3 days, shRab20 significantly reduced the expression of Rab20 in the peri-infarct region of the cortex and hippocampus compared with the shNC group (Figures 2B,C). Data from the Western blot assay demonstrated that Rab20 protein levels in the peri-infarct region of the cortex and hippocampus at 3 days after I/R were significantly increased compared with the sham group, whereas Rab20 protein levels were significantly inhibited in the I/R + shRab20 group compared with those in the I/R + shNC group (Figure 2D). To determine the effect of Rab20 inhibition on cerebral infarction, a TTC analysis of brain sections was performed. As shown in Figure 2E, mice at 3 days after I/R exhibited significantly increased infarct volume, whereas the infarct volume in the I/R + shRab20 group was significantly lower than that in the I/R + shNC group. We counted the neurological deficit score to determine the effect of Rab20 on neurological function after I/R. As shown in Figure 2F, mice at 3 days after I/R exhibited significantly worse neurological deficit score, whereas Rab20 knockdown significantly ameliorated the I/R-induced neurobehavioral deficits.

### Ras-related protein Rab-20 knockdown attenuated neuronal apoptotic death after cerebral ischemia/reperfusion

**FIGURE 3:**
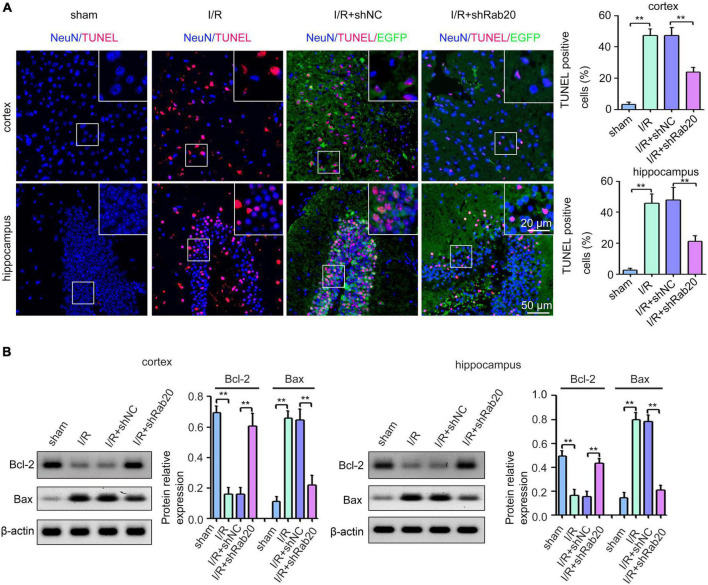
Ras-related protein Rab-20 knockdown significantly reduced I/R-induced neuronal apoptosis in mice. **(A)** Representative images and quantitative analyses of TUNEL-positive neurons in the penumbra of the cortex and hippocampus after I/R (*n* = 6). Scale bar, 20 and 50 μm. **(B)** Representative Western blot images and quantitative analyses of the apoptotic molecular markers Bcl-2 and Bax in the penumbra of the cortex and hippocampus after I/R (*n* = 6). **P < 0.01.

To determine the effect of Rab20 on cerebral I/R-induced neuronal apoptotic death, TUNEL assay was performed. Results from Figure 3A showed that TUNEL-positive neurons of peri-infarct region in cortex and hippocampus at 3 days after cerebral I/R significantly increased compared with the sham group, whereas Rab20 knockdown significantly reduced the percentage of TUNEL-positive neurons induced by cerebral I/R. The expression levels of the apoptotic molecular markers Bcl-2 and Bax were determined by Western blot at 3 days after cerebral I/R. The expression of Bcl-2 protein was significantly decreased and the expression of Bax protein was significantly increased in the peri-infarct region of cortex and hippocampus at 3 days after cerebral I/R (Figure 3B). However, Rab20 knockdown significantly reversed the decrease in Bcl-2 protein levels and the increase in Bax protein levels induced by cerebral I/R (Figure 3B).

### Ras-related protein Rab-20 knockdown attenuated oxygen-glucose deprivation/reoxygenation -induced neuronal injury in HT22 cells

**FIGURE 4:**
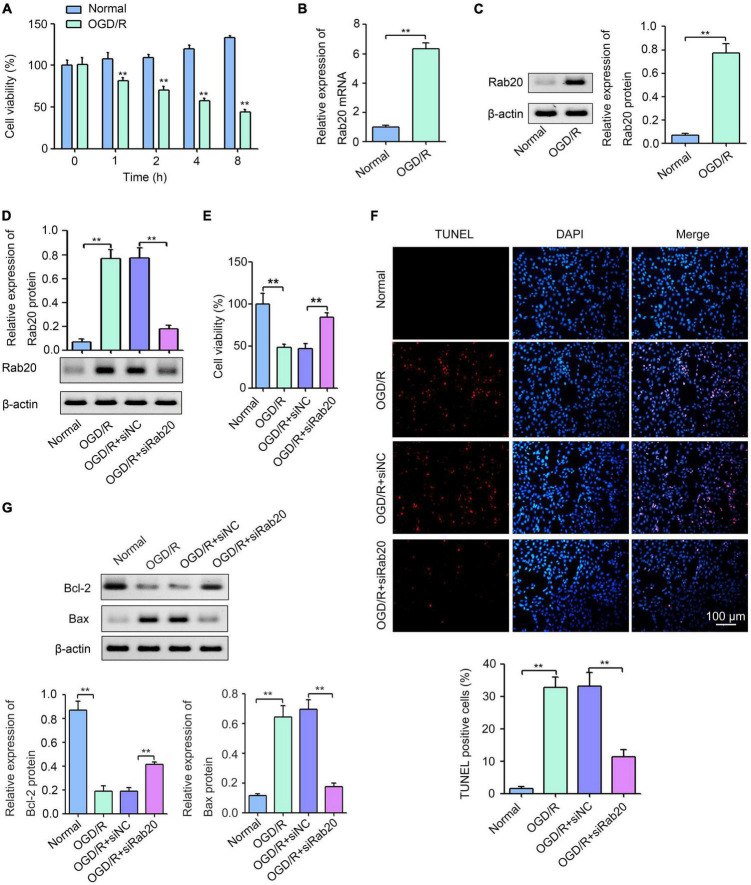
Ras-related protein Rab-20 knockdown significantly reduced OGD/R-induced neuronal injury *in vitro*. **(A)** HT22 cells were cultured in a glucose-free DMEM solution and placed into a hypoxic chamber at 1% O_2_, 5% CO_2_, and 94% N_2_ for 0, 1, 2, 4, or 8 h to mimic OGD. After OGD, cells were then given normal DMEM with 10% FBS for 24 h. CCK-8 was used to determine the cell viability (*n* = 3). **(B,C)** RT-qPCR and Western blot assays were used to determine the expression levels of Rab20 mRNA and protein in HT22 cells after OGD for 4 h and reperfusion for 24 h, respectively (*n* = 3). **(D)** HT22 cells were transfected with siRab20 and siNC for 48 h prior to OGD/R operation, and then Western blot assay was used to determine the silencing efficiency of the siRab20 in HT22 cells after OGD for 4 h and reperfusion for 24 h (*n* = 3). **(E,F)** Cell viability and cell apoptosis was detected by CCK-8 and TUNEL after OGD for 4 h and reperfusion for 24 h, respectively (*n* = 3). **(G)** Representative Western blot images and quantitative analyses of the apoptotic molecular markers Bcl-2 and Bax in HT22 cells after OGD for 4 h and reperfusion for 24 h (*n* = 3). **P < 0.01.

To further confirm the effect of Rab20 on neuronal injury after cerebral I/R, we established an OGD/R model *in vitro*. As shown in Figure 4A, HT22 cells were exposed to OGD for 0, 1, 2, 4, or 8 h, followed by reperfusion for 24 h. Cell viability was decreased with increasing OGD treatment time (Figure 4A). The cell viability of HT22 cells after OGD for 4 h was 48.0%, whereas the cell viability of HT22 cells after OGD for 2 h was 33.3%. Therefore, 4 h was selected as the optimum OGD treatment time. Western blot assay was used to determine the effect of OGD/R on Rab20 mRNA and protein levels in HT22 cells. The results showed that Rab20 mRNA and protein levels were significantly increased in the OGD/R group compared with the normal group (Figures 4B,C). To determine the role of Rab20 in neuronal injury after OGD/R, Rab20 expression was inhibited by Rab20 siRNA (Figure 4D). In addition, we found that Rab20 siRNA significantly reversed the decrease in cell viability induced by OGD/R (Figure 4E). OGD/R significantly increased apoptosis in HT22 cells, whereas Rab20 knockdown significantly inhibited OGD/R-induced apoptosis (Figure 4F). Correspondingly, the expression of Bcl-2 protein was significantly decreased, and the expression of Bax protein was significantly increased in HT22 cells after OGD/R (Figure 4G). However, Rab20 knockdown significantly reversed the decrease in Bcl-2 protein levels and the increase in Bax protein levels induced by OGD/R (Figure 4G).

### Ras-related protein Rab-20 knockdown alleviated excessive mitochondrial fission in the HT22 cells after oxygen-glucose deprivation/reoxygenation

**FIGURE 5:**
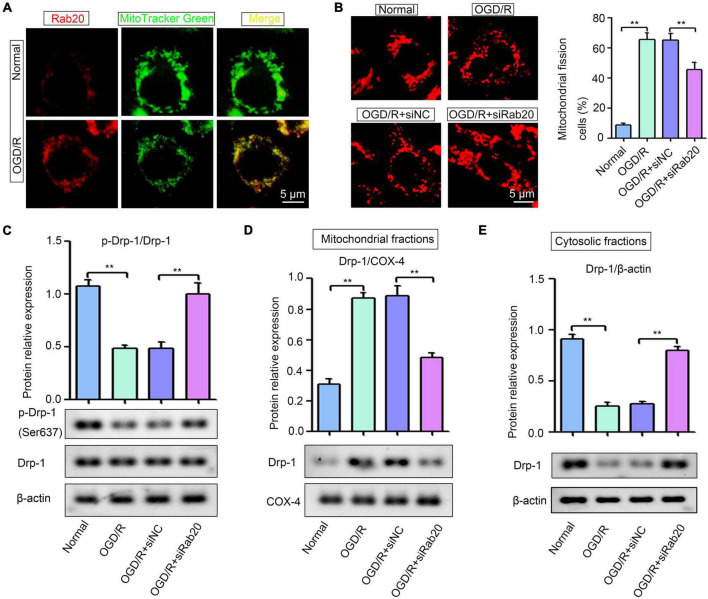
Ras-related protein Rab-20 knockdown significantly inhibited mitochondrial fission in HT22 cells after OGD/R. **(A)** HT22 cells were transfected with siRab20 and siNC for 48 h prior to OGD/R operation, and then double immunofluorescence staining for Rab20 (red) in mitochondria (MitoTracker Green, green) was performed in HT22 cells after OGD for 4 h and reperfusion for 24 h. Scale bar, 5 μm. **(B)** MitoTracker^®^ Deep Red FM staining was used to determine the mitochondrial morphology (*n* = 3). Scale bar, 5 μm. **(C)** Total levels of Drp-1 and p-Drp1 (Ser637) were determined by Western blot (*n* = 3). **(D,E)** Drp-1 expression levels in the mitochondrial and cytosolic fractions were determined by Western blot (*n* = 3). **P < 0.01.

Ras-related protein Rab-20 has been shown as a predominant mitochondrial protein (Hackenbeck et al., 2011). Thus, we determined whether increased Rab20 expression induced by OGD/R was mainly located in mitochondria in HT22 cells. Figure 5A shows that mitochondria were visualized by the mitochondrial marker MitoTracker Green, and Rab20 protein was stained with a Rab20 antibody (red). All cells showed a colocalization with labeled mitochondria and Rab20 (Figure 5A). Rabs and Rab effectors have been implicated in mitochondrial fission (Farmer and Caplan, 2020). After stroke, mitochondrial fission was induced to increase mitochondrial energy production for the maintenance of neural function, whereas excessive mitochondrial fission was detrimental to neurons (Andrabi et al., 2019). Thus, we determined whether increased Rab20 expression was associated with mitochondrial fission. As shown in Figure 5B, mitochondria in the HT22 cells had an elongated tubular structure in the normal group, whereas OGD/R treatment caused punctuated structures in HT22 cells. The proportion of fragmented mitochondria was increased in HT22 cells after OGD/R, whereas Rab20 knockdown significantly reduced OGD/R-induced mitochondrial fission in HT22 cells (Figure 5B). Rab proteins are involved in dynamin-related protein 1 (Drp-1)-mediated mitochondrial fragmentation (Landry et al., 2014; Liang et al., 2020). Thus, we determined whether Rab20 alleviated OGD/R-induced mitochondrial fission by mediating Drp-1. Drp-1 phosphorylation at Ser637 decreased in HT22 cells after OGD/R (Figure 5C). As shown in Figures 5D,E, OGD/R induced the location of Drp-1 at mitochondria and inhibited the location of Drp-1 at cytoplasm. However, Rab20 knockdown significantly attenuated the location of Drp-1 at mitochondria and the inhibition of Drp-1 phosphorylation at Ser637 induced by OGD/R (Figures 5C–E).

### Ras-related protein Rab-20 knockdown improved mitochondrial dysfunction in HT22 cells after oxygen-glucose deprivation/reoxygenation

**FIGURE 6:**
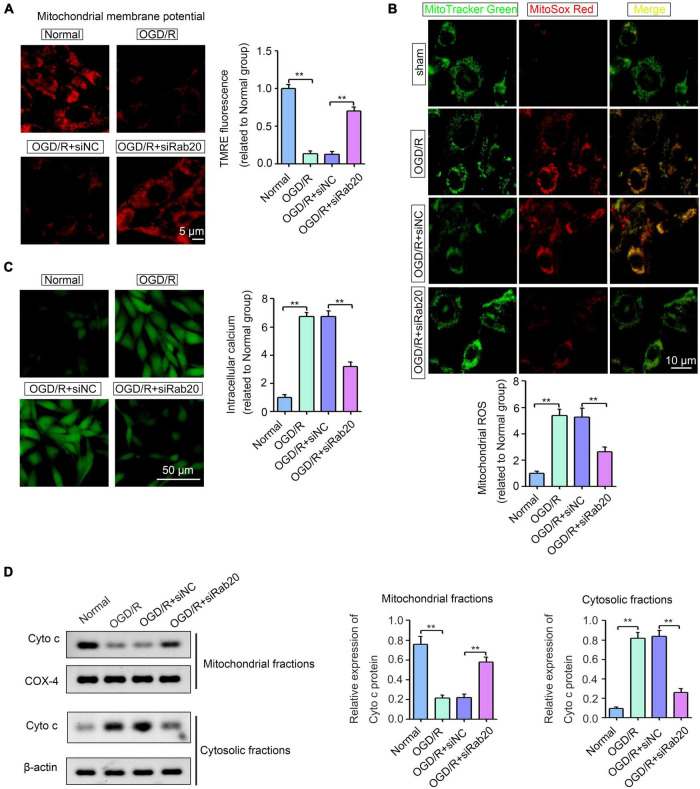
Ras-related protein Rab-20 knockdown significantly ameliorated mitochondrial dysfunction in HT22 cells after OGD/R. **(A)** HT22 cells were transfected with siRab20 and siNC for 48 h prior to OGD/R operation, and then TMRE staining was used to analyze mitochondrial membrane potential (Δψm) in HT22 cells after OGD for 4 h and reperfusion for 24 h. Scale bar, 5 μm. **(B)** Mitochondrial-derived ROS was stained by MitoSox Red and MitoTracker Green (*n* = 3). Scale bar, 10 μm. **(C)** Fluo-3AM staining was used to determine the intracellular calcium accumulation (*n* = 3). Scale bar, 50 μm. **(D)** Cyto c expression levels in the mitochondrial and cytosolic fractions were determined by Western blot (*n* = 3). **P < 0.01.

Ras-related protein Rab-20 knockdown significantly alleviated excessive mitochondrial fission. Thus, we further determined the effect of Rab20 on mitochondrial dysfunction, as indicated by mitochondrial membrane potential (Δψm) collapse, excessive ROS production, cellular calcium accumulation and Cyto c release. To measure mitochondrial membrane potential (Δψm) collapse, TMRE staining was performed. As shown in Figure 6A, TMRE signal was significantly inhibited in HT22 cells after OGD/R, whereas Rab20 knockdown significantly reversed the inhibition of TMRE signal induced by OGD/R. To measure mitochondrial-derived ROS, HT22 cells were stained by MitoSox Red and MitoTracker Green. The mitochondrial-derived ROS was significantly increased in HT22 cells after OGD/R, whereas Rab20 knockdown significantly reversed OGD/R-induced ROS production (Figure 6B). Fluo-3AM staining showed that cellular calcium accumulation was significantly increased in HT22 cells after OGD/R, whereas this alteration was significantly reversed by Rab20 knockdown (Figure 6C). As shown in Figure 6D, OGD/R treatment significantly induced Cyto c release from the mitochondria into the cytosol, whereas this alteration was significantly reversed by Rab20 knockdown.

## Discussion

Ras-related protein Rab-20 is among the Rab family of small GTPases, which are involved in membrane traffic in all eukaryotic cells (Stenmark, 2009). Interestingly, Rab20 was induced under stress. For example, Rab20 expression was increased in retinal endothelial cells and retinal Müller cells exposed to high glucose (Kim et al., 2020). In a hypoxic microenvironment, HIF-1 upregulation induced Rab20 expression (Görgens et al., 2017). In the present study, we found for the first time that Rab20 was significantly increased in the injured hemisphere after I/R (Figures 1A,B), and elevated Rab20 was mainly expressed in neurons but not in astrocytes and microglia (Figure 1C). Consistent with our findings, Rab20 was upregulated during the acute phase of inflammation in mice (Liang et al., 2012). However, upregulated Rab20 was observed in active microglial cells during the acute phase of inflammation (Liang et al., 2012). Thus, Rab20 may show diverse cellular localization under different pathological conditions.

Ras-related protein Rab-20 was reportedly involved in stress-induced apoptosis (Hackenbeck et al., 2011; Kim et al., 2020). For instance, upregulated Rab20 induced by high glucose contributed to cell apoptosis in retinal endothelial cells and retinal Müller cells (Kim et al., 2020). Rab20 induced by HIF-1 also contributed to hypoxia-induced apoptosis (Hackenbeck et al., 2011). Whether the neuronal injury was influenced by Rab20 under cerebral I/R was unknown. Here, we found that Rab20 knockdown caused by injecting shRab20 AAV particles into the mouse brain significantly ameliorated the neurological outcome (Figures 2E,F) and reduced I/R-induced neuronal apoptosis, which was stained intensively by the TUNEL reaction (Figure 3A). To further verify the role of Rab20 in I/R-induced cell apoptosis, we assessed the effect of Rab20 on several key apoptosis-associated signal proteins including Bax and Bcl-2. As expected, Rab20 knockdown significantly reversed the decrease in Bcl-2 protein levels and the increase in Bax protein levels induced by cerebral I/R (Figure 3B). To further confirm the effect of Rab20 on neurological injury *in vitro*, we established an OGD/R model. Rab20 expression was significantly increased in HT22 cells after OGD/R (Figures 4B,C), and Rab20 inhibition by siRNA significantly ameliorated OGD/R-induced inhibition of cell viability (Figure 4E) and apoptosis (Figure 4F) in the HT22 cells. Moreover, Rab20 knockdown significantly restored the decrease in Bcl-2 protein levels and the increase in Bax protein levels induced by OGD/R (Figure 4G). These results further supported the proapoptotic action of Rab20 in I/R-induced neuronal injury.

A previous study has reported that Rab20 mainly colocalizes with mitochondria in HeLa and HKC-8 cells (Hackenbeck et al., 2011). We showed that upregulated Rab20 protein was mainly located in mitochondria after OGD/R (Figure 5A). Mitochondria are the powerhouse of the cell and organized in a highly dynamic tubular network characterized by fusion and fission (Cardoso et al., 2010). Mitochondrial dynamics is essential for maintaining the normal physiological function of cells through continuous fusion-division. However, excessive mitochondrial fission affects energy metabolism in cells, and induces apoptosis after ischemic stroke (Doyle et al., 2008). Rabs and Rab effectors are associated with mitochondrial fission (Farmer and Caplan, 2020). For instance, Rab7 can mark the mitochondria for fission by promoting contact sites between lysosomes and mitochondria (Wong et al., 2018). Rab32, a mitochondrial PKA anchoring protein, is involved in the assembly of mitochondrial fission complex (Alto et al., 2002). These studies led to the hypothesis that Rab20 may affect the I/R-induced neuronal injury by mediating mitochondrial fission. As expected, our data showed that Rab20 knockdown significantly reduced OGD/R-induced mitochondrial fission in HT22 cells (Figure 5B). Drp-1 plays a crucial role in regulating mitochondrial fission. Under homeostatic states, Drp-1 is allocated in the cytoplasm, whereas during ischemic stroke, Drp-1 is activated by dephosphorylation at Ser637, thereby promoting the recruitment of Drp-1 to the mitochondria *via* its receptor proteins, mitochondrial fragmentation, and exacerbated apoptotic cell death after ischemic stroke (Flippo et al., 2020; Tian et al., 2022). Rabs are involved in Drp-1-mediated mitochondrial fragmentation (Landry et al., 2014; Liang et al., 2020). For example, Rab11a regulates Drp1-mediated fission by promoting the stable association of Drp-1 with mitochondrial membranes (Landry et al., 2014). These important observations have promoted further exploration of the interaction between Rab20 and Drp-1 in mitochondrial fragmentation after ischemic stroke. Our results showed that Rab20 knockdown significantly ameliorated the inhibition of Drp-1 phosphorylation at Ser637 induced by OGD/R *in vitro* (Figure 5C). In addition, Rab20 inhibition significantly reversed the mitochondrial Drp1 recruitment induced by OGD/R in HT22 cells (Figures 5D,E). Thus, Rab20 may promote mitochondrial fragmentation by inhibiting Drp-1 phosphorylation at Ser637, thereby inducing mitochondrial Drp1 recruitment and neuronal apoptosis after ischemic stroke.

When mitochondrial fission is induced, the number of dysfunctional mitochondria in neurons increases after I/R (Yang et al., 2018). Mitochondrial dysfunction, which can lead to mitochondrial membrane potential (ΔΨm) collapse, leads to the overproduction of ROS, calcium accumulation and Cyto c release, and promotes neural apoptosis in ischemic stroke (Andrabi et al., 2020). The interaction between calcium overload, ROS production, and the mitochondrial permeability transition pores (MPTP) leads to the increase in mitochondrial fission and apoptotic death in ischemic stroke (Andrabi et al., 2019). Our results showed that Rab20 knockdown significantly alleviated mitochondrial membrane potential (Δψm) collapse, the accumulation of mitochondrial-derived ROS and cellular calcium induced by OGD/R in HT22 cells (Figures 6A–C). In addition, Rab20 knockdown significantly reversed Cyto c release from mitochondria to cytosol, which was induced by OGD/R in HT22 cells (Figure 6D). Increasing evidence indicate that mitochondrial events, such as ROS production and cellular calcium accumulation, leads to Cyto c release from the mitochondria to the cytosol, thereby promoting apoptotic cascade during ischemic stroke (Andrabi et al., 2019). Thus, targeting Rab20 may be a novel approach for alleviating the mitochondrial dysfunction induced by I/R.

## Conclusion

In summary, Rab20 expression was increased in neurons after I/R, and upregulated Rab20 induced mitochondrial fission and dysfunction, which in turn caused neuronal apoptosis in I/R injury.

## Data availability statement

The original contributions presented in this study are included in the article/Supplementary material, further inquiries can be directed to the corresponding author.

## Ethics statement

The animal study was reviewed and approved by the Ethics Committee of Lanzhou University Second Hospital.

## Author contributions

JG and JL conceived and designed the study. JG, JL, YB, and WL performed the experiments. JG, YB, LZ, and JH analyzed the data. JG and YB wrote and revised the manuscript. All authors read and approved the final manuscript.
