# Supplementary material for: Ras-related protein Rab-20 inhibition alleviates cerebral ischemia/reperfusion injury by inhibiting mitochondrial fission and dysfunction
Source: Front Mol Neurosci. 2022 Oct 25;15:986710. doi: 10.3389/fnmol.2022.986710 (PMC9640763; doi:10.3389/fnmol.2022.986710)
Supplement: Supplementary file 1 [file Data_Sheet_1.PDF]

Figure 1B

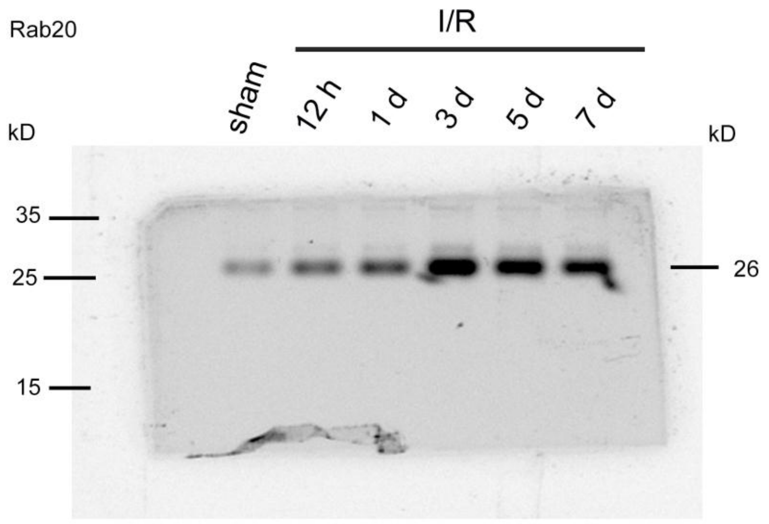
Rab20
I/R
sham
12 h
1 d
3 d
5 d
7 d
kD
kD
35
25
15
26

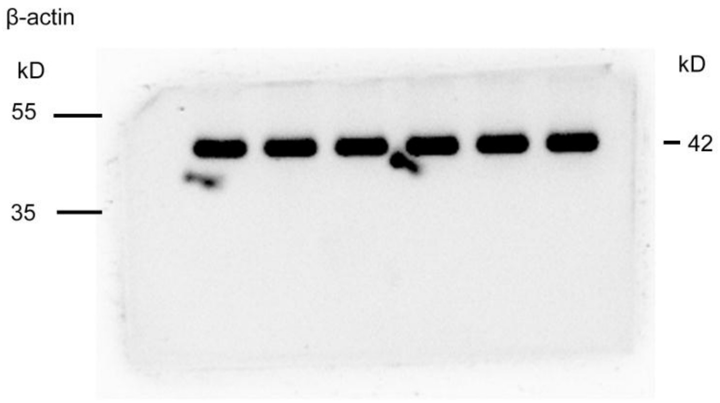
β-actin
kD
kD
55
35
42

Figure 2D

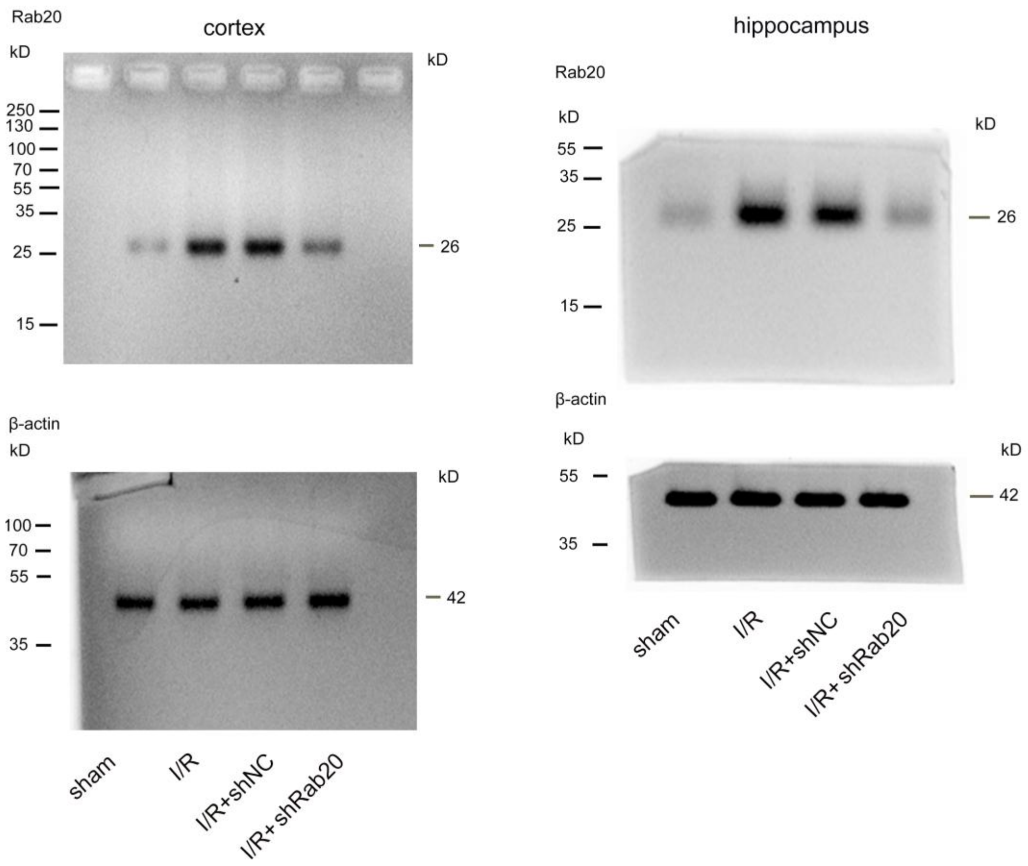

Rab20
cortex
kD
kD
250
130
100
70
55
35
25
15
26
β-actin
kD
kD
100
70
55
35
42
sham
I/R
I/R+shNC
I/R+shRab20
hippocampus
Rab20
kD
kD
55
35
25
15
26
β-actin
kD
kD
55
35
42
sham
I/R
I/R+shNC
I/R+shRab20

Figure 3B

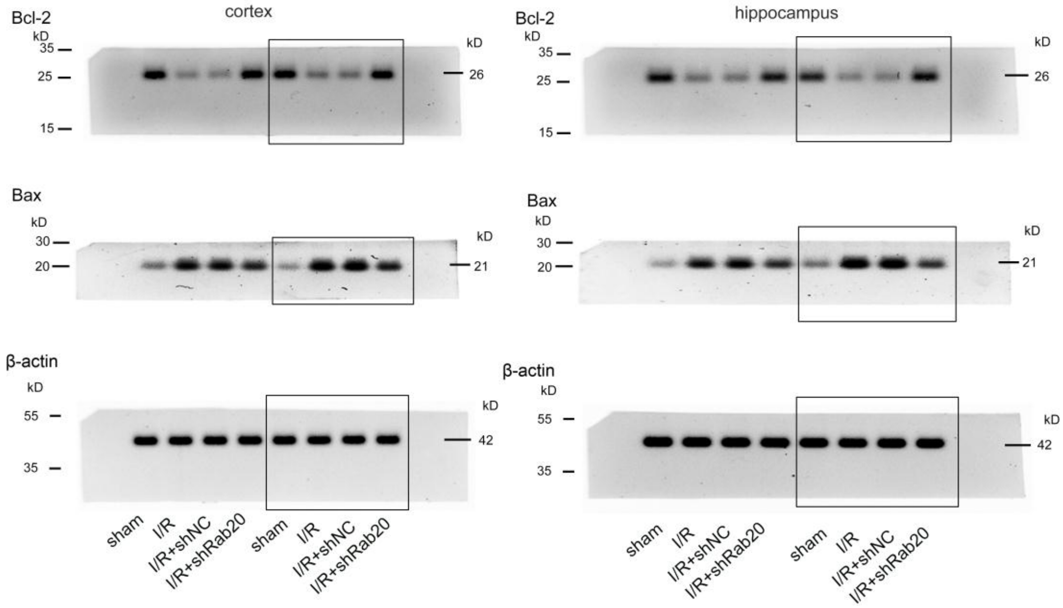
cortex
hippocampus
Bcl-2
kD
35
25
15
26
Bax
30
20
21
β-actin
55
35
42
sham
I/R
I/R+shNC
I/R+shRab20

Figure 4C,D,G

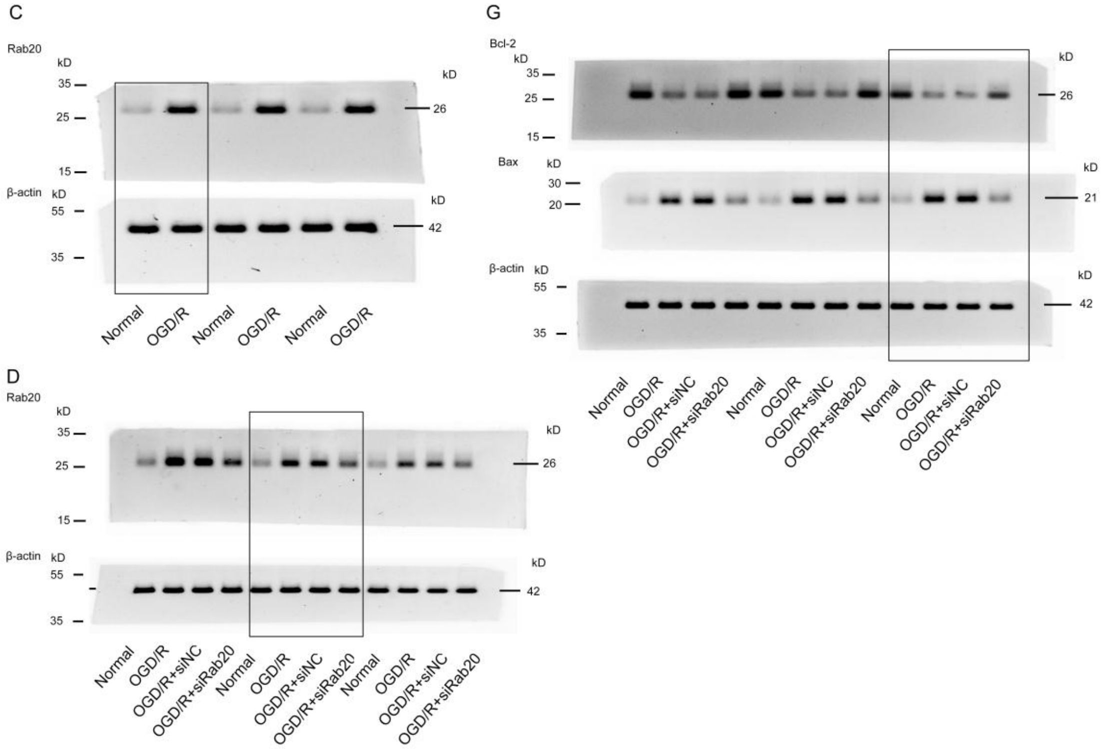
C
Rab20
kD
35
25
15
kD
26
β-actin
kD
55
35
kD
42
Normal
OGD/R
Normal
OGD/R
Normal
OGD/R
D
Rab20
kD
35
25
15
kD
26
β-actin
kD
55
35
kD
42
Normal
OGD/R
OGD/R+siNC
OGD/R+siRab20
Normal
OGD/R
OGD/R+siNC
OGD/R+siRab20
Normal
OGD/R
OGD/R+siNC
OGD/R+siRab20
G
Bcl-2
kD
35
25
15
kD
26
Bax
kD
30
20
kD
21
β-actin
kD
55
35
kD
42
Normal
OGD/R
OGD/R+siNC
OGD/R+siRab20
Normal
OGD/R
OGD/R+siNC
OGD/R+siRab20
Normal
OGD/R
OGD/R+siNC
OGD/R+siRab20

Figure 5C-E

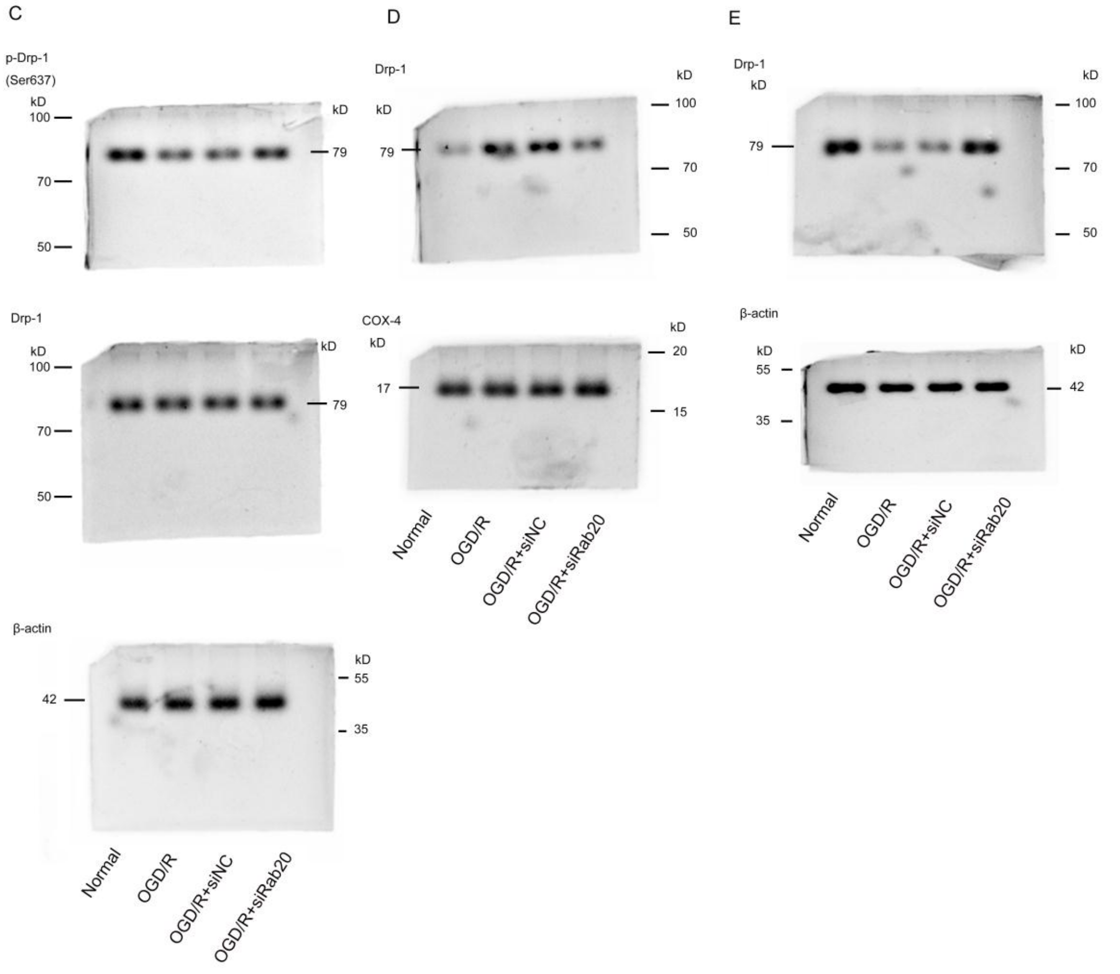
C
p-Drp-1
(Ser637)
kD
100
70
50
kD
79
Drp-1
kD
100
70
50
kD
79
β-actin
42
kD
55
35
Normal
OGD/R
OGD/R+siNC
OGD/R+siRab20
D
Drp-1
kD
79
kD
100
70
50
COX-4
kD
17
kD
20
15
Normal
OGD/R
OGD/R+siNC
OGD/R+siRab20
E
Drp-1
kD
79
kD
100
70
50
β-actin
kD
55
35
kD
42
Normal
OGD/R
OGD/R+siNC
OGD/R+siRab20

Figure 6D

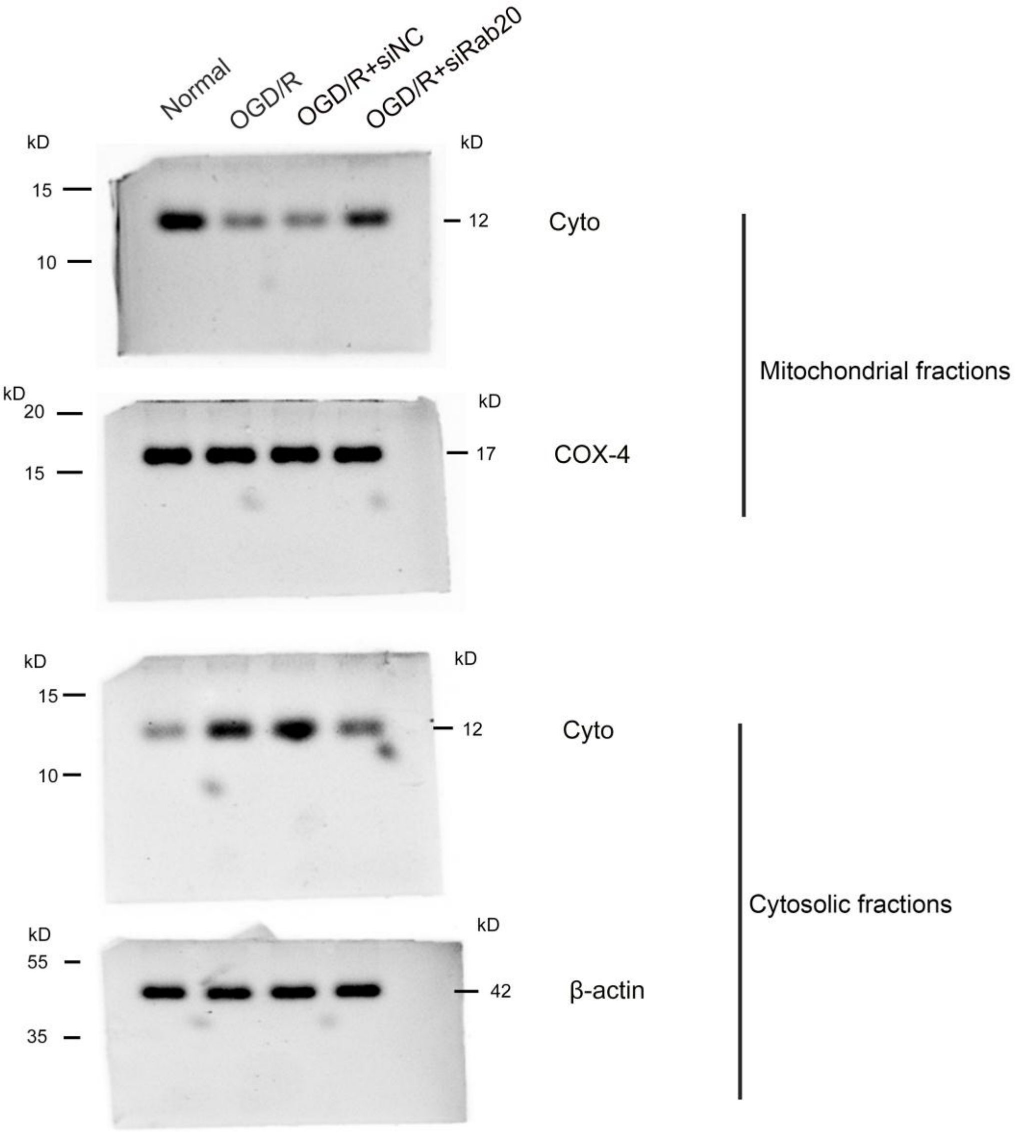

Normal
OGD/R
OGD/R+siNC
OGD/R+siRab20
kD
15
10
kD
12
Cyto
kD
20
15
kD
17
COX-4
Mitochondrial fractions
kD
15
10
kD
12
Cyto
kD
55
35
kD
42
β-actin
Cytosolic fractions
